# Metastatic Leiomyosarcoma Involving Bone and Kidney in an Adult Budgerigar (*Melopsittacus undulatus*)

**DOI:** 10.1002/vms3.71199

**Published:** 2026-08-24

**Authors:** Hemad Shafiei, Reza Kheirandish, Hamzeh Soltani, Ali Keshavarzrad, Shahriar Dabiri

**Affiliations:** ^1^ Department of Clinical Science, Faculty of Veterinary Medicine Shahid Bahonar University of Kerman Kerman Iran; ^2^ Department of Pathobiology, Faculty of Veterinary Medicine Shahid Bahonar University of Kerman Kerman Iran; ^3^ Doctor of Veterinary Medicine, Faculty of Veterinary Medicine Shahid Bahonar University of Kerman Kerman Iran; ^4^ Department of Pathology, Pathology and Stem Cell Research Center, Afzalipour Faculty of Medicine Kerman University of Medical Sciences Kerman Iran

**Keywords:** alpha‐smooth muscle actin (α‐SMA), budgerigar, desmin, histopathology, leiomyosarcoma, vimentin

## Abstract

Leiomyosarcoma (LMS), a malignant neoplasm of smooth muscle cell origin, is relatively rare in birds. This study presents the occurrence of this tumour in a 2‐year‐old male budgerigar. The bird was brought to the Ashian Veterinary Hospital with complaints of swelling in the medial area of the left leg. Clinical examination and radiological imaging confirmed the presence of soft tissue swelling in the medial region of the left leg. Empirical antibiotic and anti‐inflammatory therapy were used to distinguish infectious inflammation from the tumour mass. After 12 days, a clinical diagnosis of suspected neoplasm was made due to a lack of improvement and increasing mass size. Given the deterioration of the bird's general condition, euthanasia was recommended and performed. At necropsy, all viscera were biopsied for histopathological evaluation. Microscopic examinations confirmed the initial diagnosis of LMS. To confirm the diagnosis, immunohistochemical staining was performed using antibodies to alpha‐smooth muscle actin (α‐SMA), vimentin and desmin. The results of α‐SMA and desmin staining definitively confirmed the presence of an LMS neoplasm with metastasis to the bone and kidneys; however, vimentin staining was negative.

## Graphical abstract

A 2‐year‐old male budgerigar presents with swelling of the medial left leg. Soft tissue neoplasia is suspected after empirical treatment fails. Histopathology and immunohistochemistry (α‐SMA and desmin positivity, vimentin negativity) confirm leiomyosarcoma, with metastasis to bone and kidneys. This case documents uncommon metastatic spread in an avian species.

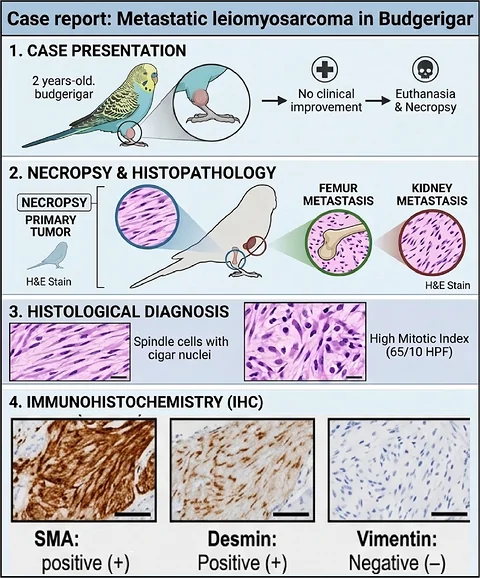

## 1 Introduction

The incidence of neoplasia varies significantly among bird species and is influenced by genetic factors, environmental exposures and management protocols (Latimer 1994). Advances in veterinary medicine in breeding, health and treatment have increased the lifespan of domestic birds, which in turn has led to an increase in the incidence of age‐related disorders, including neoplastic processes (Robat et al. 2017). Leiomyosarcoma (LMS) is a rare malignant neoplasm that can be seen in aging pet birds (Schmidt et al. 2024). This tumour originates from smooth muscle cells and typically spreads to nearby tissues, rarely to other organs. LMS may occur in several organs; however, it most commonly occurs in the spleen, gastrointestinal system and reproductive tract (Rasche et al. 2023; Timurkaan et al. 2016). The tumour is confirmed by histopathological and immunohistochemical findings, which often include staining for desmin, smooth muscle actin (SMA) and vimentin (Timurkaan et al. 2016). This article reports a case of LMS in a budgerigar with metastasis to the kidney and bone.

## 2 Case Description

**FIGURE 1:**
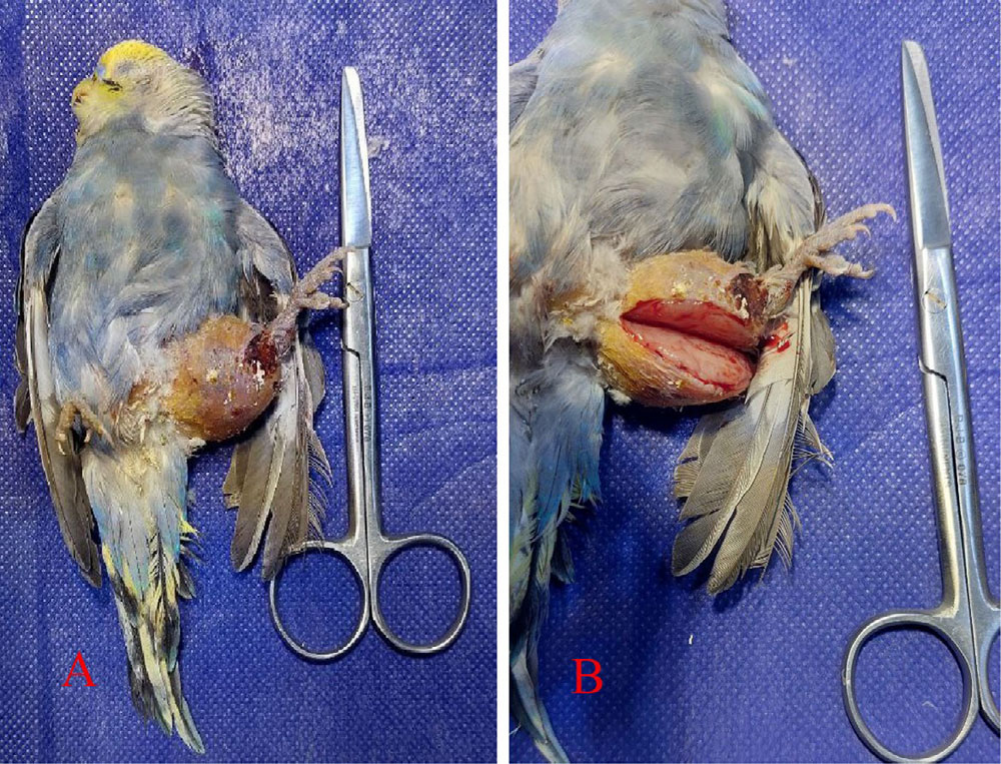
Postmortem appearance of neoplastic mass. (A) Spherical tumour tissue with a diameter of about 3 cm in the medial region of the left leg. (B) The cut surface revealed a soft and cream‐coloured mass.

**FIGURE 2:**
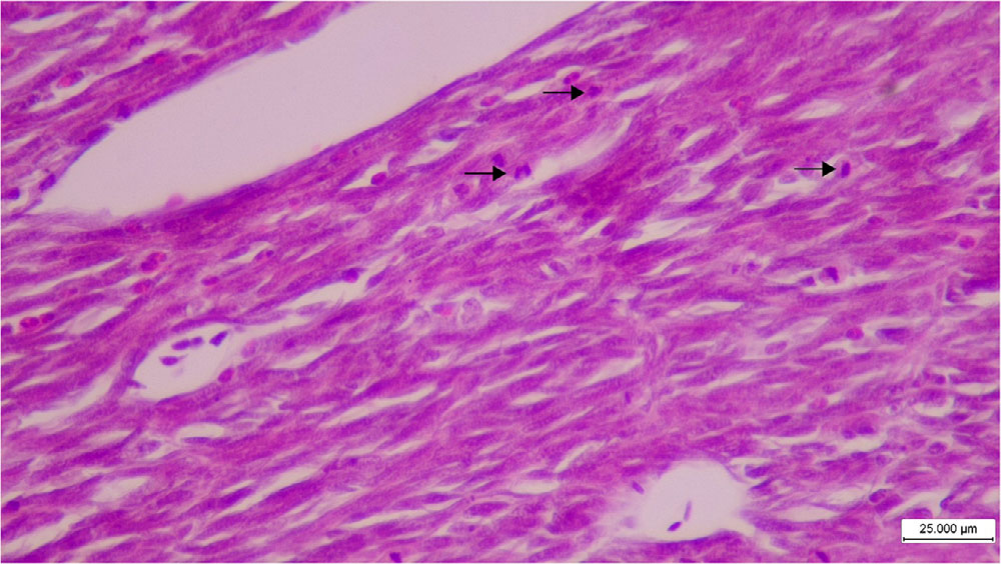
Histopathological examination diagnosing leiomyosarcoma in a budgerigar. Neoplastic tissue: This micrograph shows mildly pleomorphic, cigar‐shaped tumoral cells that are arranged in intersecting fascicles. Numerous mitotic figures (arrows) are seen. H&E, Bar = 25.000 µm.

**FIGURE 3:**
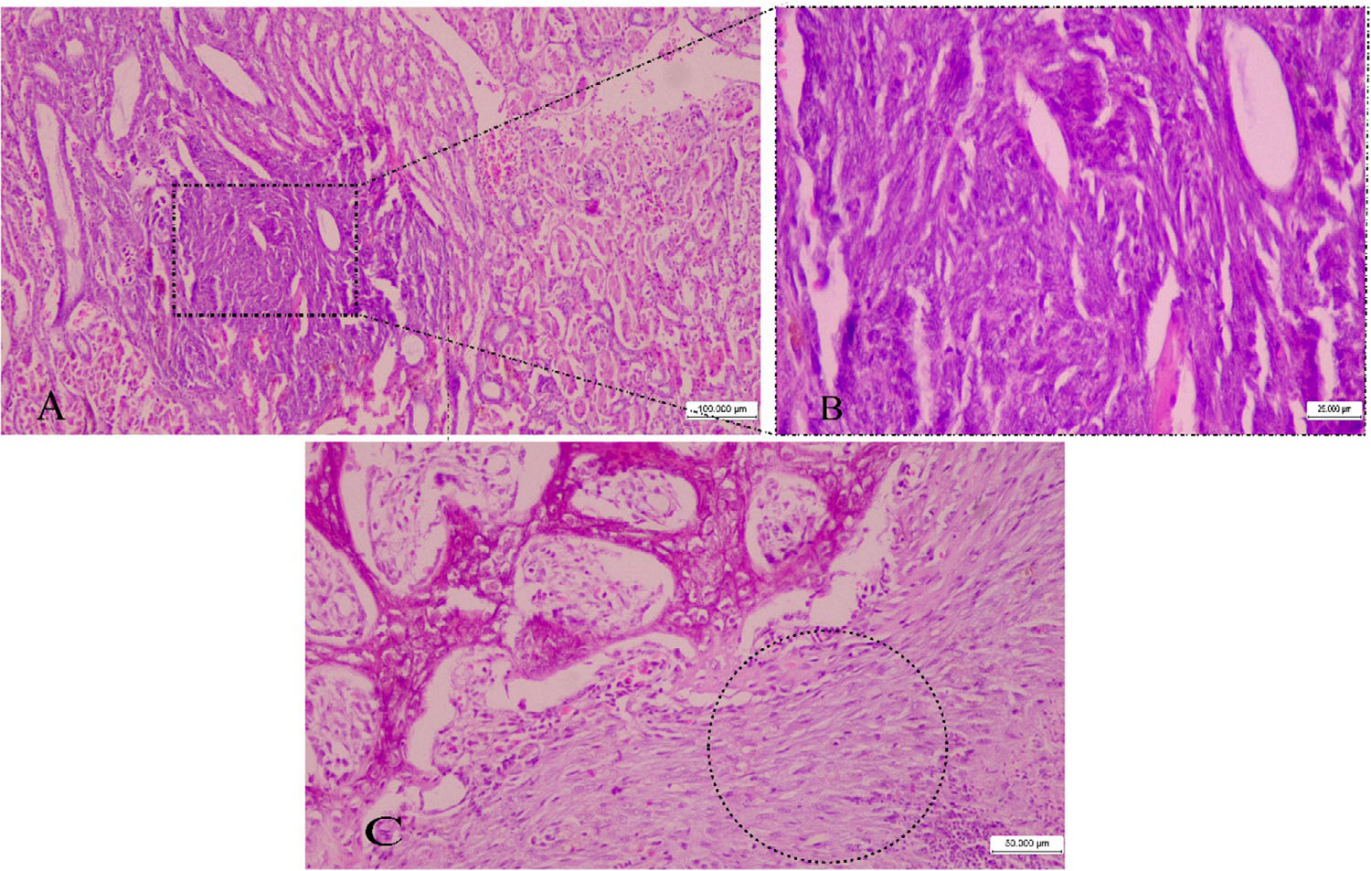
Histopathological examination diagnosing leiomyosarcoma in a budgerigar. (A) Kidney neoplastic tissue. H&E, Bar = 100.000 µm. (B) Invasive tumoral mass in the kidney. H&E, Bar = 25.000 µm. (C) Bone tissue: invasive tumoral mass (circle) in the femur bone. H&E, Bar = 50.000 µm.

**FIGURE 4:**
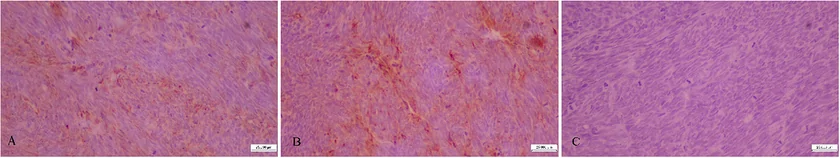
Immunohistochemical stains diagnosing leiomyosarcoma in a budgerigar. (A) Tumoral cells show positive immunoperoxidase staining for desmin. IHC, Bar = 25.000 µm. (B) Positive SMA staining is seen. IHC, Bar = 25.000 µm. (C) Negative vimentin staining is seen. IHC, Bar = 25.000 µm.

A 2‐year‐old male budgerigar (*Melopsittacus undulatus*), weighing 32 g and exhibiting a substantial mass in the medial region of the left leg, was referred to a specialized Ashian veterinary hospital for evaluation. A preliminary diagnostic evaluation was conducted to distinguish between infectious tissue inflammation and a soft‐tissue tumour (such as LMS), which involved starting antibiotic and anti‐inflammatory therapies using 10% enrofloxacin (Enrofloxacin 10% Rooyan, Rooyan Darou, Iran) and meloxicam (Melocox 7.5 mg, Chemidarou Industrial Company, Iran). The recommended dosage of enrofloxacin (150 mg/L) in drinking water was administered every 24 h for 10 days, while meloxicam was administered at 0.5 mg/kg PO every 24 h for 3 days. Even with treatment, the mass continued to grow, and no signs of clinical improvement were observed. Considering the absence of response to antibiotic and anti‐inflammatory treatments, along with the aggressive growth characteristics of the mass, an initial diagnosis of a tumour was established. Because the bird was in very poor physical condition and showed extreme lethargy, euthanasia was carried out, and the bird was sent for an autopsy. After euthanasia, a complete necropsy was performed with the owner's consent. Tissue samples from all major organs and the mass were collected for histopathological analysis. A spherical tumour, approximately 3 cm in diameter, with a soft consistency and cream‐coloured appearance, was observed in the medial region of the left leg (Figure 1). All samples were fixed in 10% neutral buffered formalin, sent to the pathology laboratory and the histopathological results are as follows. Histologically, the tumour mass was composed of mildly pleomorphic, spindle and blunt‐ended cells (cigar‐shaped nuclear). Tumour cells showed mild atypia, and the mitotic count was 65 per 10 high‐power fields (10HPF) (Figure 2). In some sections, tumour cells invaded the adjacent bone (femur) (Figure 3A). Metastasis to the kidney was present (Figure 3B). Immunochemistry staining was positive for desmin and SMA (Figure 4A,B), but it was negative for vimentin (Figure 4C). Histologic characteristics and immunochemistry results were consistent with LMS.

## 3 Discussion

The occurrence of neoplasms is considered a major challenge to the health of ornamental birds, especially small species such as budgerigars. Although accurate and systematic statistics on the epidemiology of tumours in different bird populations, similar to those in human medicine, are not available, data from routine autopsies and case reports indicate the prevalence of this group of diseases (Latimer 1994; Sánchez‐Godoy et al. 2020). Neoplasia is more commonly reported in companion and aviary birds than in free‐ranging birds due to closer observation, longer lifespans and possible genetic predisposition from inbreeding. Most reports of neoplasia involve captive birds, especially budgerigars, with an incidence ranging from 16.8% to 24.2%. In a veterinary lab with diverse avian cases, budgerigars accounted for 69.7% of psittacine neoplasms and 41% of all avian neoplasms. The overall incidence of neoplasia was around 3.8% in all avian submissions (Latimer 1994). In general, birds, like mammals, are susceptible to a wide range of benign and malignant tumours of epithelial, mesenchymal, and mixed origin (Schmidt et al. 2024). The distribution and relative frequency of these tumours can be influenced by several factors, including species, breed, age, sex, environmental conditions, and genetic factors. For example, genital tumours are particularly prevalent in older female psittacine birds, while cutaneous and subcutaneous tumours can be observed in a wide range of species (Latimer 1994; Rasche et al. 2023; Sánchez‐Godoy et al. 2020). Retrospective studies conducted on autopsy collections have listed lipomas, fibromas, fibrosarcomas, various adenocarcinomas (especially in the liver and intestine), ovarian and testicular tumours, as well as kidney‐related neoplasms as among the most common tumours of this type (Robat et al. 2017).

LMS is a malignant neoplasm of smooth muscle cell origin, classified as a rare tumour in avian pet species (Robat et al. 2017; Fabian et al. 2021). This tumour can occur in any tissue or organ containing smooth muscle, including the walls of the gastrointestinal tract and uterus, as well as blood vessels and the spleen. There are sporadic reports of LMS in various bird species, including cockatoos, African grey parrots and, in particular, budgerigars in the scientific literature. However, in cases where the tumour is poorly differentiated, it can be complicated and sometimes impossible to distinguish it from other spindle cell sarcomas, such as fibrosarcoma, histosarcoma, and even schwannoma (Sutherland et al. 2016). In such circumstances, the IHC technique serves as a diagnostic necessity to confirm the origin of the cell line and reach a definitive diagnosis (Robat et al. 2017; Sutherland et al. 2016; LaRock and Ginn 1997).

Case studies in budgerigars and related species have shown that muscle fasciculus tumours can present both focally (e.g., cutaneous or oral) and viscerally, and in some cases metastasize to adjacent tissues or distant organs, although the frequency and exact pattern of metastasis in birds is not yet well established (Timurkaan et al. 2016). A review of human and veterinary studies suggests that bone and, rarely, the kidney can be considered as secondary sites for LMS, although in humans the most common distant sites are usually lung, liver and bone, and the kidney has been reported less frequently as a primary or secondary site (LiBrizzi et al. 2022).

The metastatic behaviour of LMS tumours in birds is generally rare; however, there have been reported cases of metastasis to various organs, including the liver, lungs, kidneys and spleen. Besides systemic circulation, birds may also experience metastasis through specific anatomical pathways, such as the renal‐portal shunt. This shunt directs blood from the hindlimbs to the liver before it enters general circulation, potentially resulting in the accumulation of tumour cells in these regions (Frazier et al. 1993). Furthermore, the biological characteristics of birds, notably the absence of large, organized lymph nodes, which diminishes their lymphatic filtering capacity, might significantly influence patterns of metastasis (Latimer 1994). Some studies indicate that LMS tumours exhibiting a lower degree of differentiation, as well as myxoid or dedifferentiated features, are more prone to metastasize. This tendency may be linked to genetic alterations and cellular transdifferentiation that enhance the tumour's invasiveness and propensity to spread (Schmidt et al. 2024). Numerous studies indicate that metastasis in avian LMSs frequently manifests at advanced stages of the disease, typically following the initial progression of local tumour growth. Consequently, early diagnosis and prompt intervention are critically important for curtailing disease dissemination (Fabian et al. 2021; Kheirandish et al. 2016).

The immunophenotypic profile of vimentin as a mesenchymal marker is essential in diagnosing mesenchymal tumours, including LMSs. Vimentin, an intermediate filament protein, is commonly expressed in mesenchymal cells, with higher levels often associated with malignancy and tumour progression. Studies on avian LMSs have shown a positive reaction to vimentin, suggesting a mesenchymal origin; however, as indicated in Table 1, the expression intensity can vary, with some cases showing weak or negative results. The differences observed may be attributed to the level of tumour differentiation or variations in protein expression across different species. Additionally, vimentin, when analysed alongside smooth muscle alpha‐actin and desmin, serves as a definitive marker to differentiate LMS from other spindle‐shaped tumours (Cardoso and Levy 2014).

**TABLE 1:** Comparison of reported cases of leiomyosarcoma in budgerigars and other birds.

Metastasis	Species	Age	Sex	Tumour	Location	IHC	References
No	Budgerigar	2 years	Male	Leiomyosarcomas	Beak	α‐SMA (Positive) S100 (Negative) CK AE1/AE3 (Lacked) PNL2 (Lacked) Melan‐A (Lacked)	(Rasche et al. 2023)
No	Budgerigar	6 years	Female	Leiomyosarcoma	Caudal abdominal region	Desmin (Positive) α‐SMA (Positive)	(Shivaprasad and Palmieri 2012)
No	Cockatiels	5 years	NS	Leiomyosarcoma	Wing	α‐SMA (Positive) Vimentin (Positive)	(Bonel et al. 2019)
No	Cockatiel	11 years	Female	Myxoid Leiomyosarcoma	Oviduct and Uterus	Desmin (Positive) α‐SMA (Positive) Vimentin (Negative)	(Golchin and Borhanikiya 2024)
No	White Carneau Pigeon	—	NS	Leiomyosarcoma	Pulmonary	Desmin (Positive) Actin (Positive)	(Newman and West 2001)
No	Pouter Pigeon	Aged	Male	Leiomyosarcoma	Ileal	Desmin (Positive) α‐SMA (Positive) Cytokeratin (Negative) c‐kit. (Negative)	(Gornatti Churria and Loukopoulos 2018)
No	Zebra Finch	Adult	Male	Leiomyosarcoma	Intestinal	Desmin (Positive) α‐SMA (Positive) Vimentin (Positive) KIT (Negative)	(Cardoso and Levy 2014)
Bone	Budgerigar	5 years	Female	Cutaneous Leiomyosarcoma	Wing	Desmin (Positive) α‐SMA (Positive) Vimentin (Positive) S100 (Negative) CD68 (Negative)	(Timurkaan et al. 2016)
Spleen, lung, kidney, liver	Budgerigar	Adult	Male	Leiomyosarcoma	Hind limb	α‐SMA (Positive)	(Patial et al. 2020)
Multiple viscera	Domestic Pigeon	4 years	NS	Cutaneous Leiomyosarcoma	Wing	α‐SMA (Positive) Vimentin (Positive)	(Stenzel et al. 2017)
Pulmonary system, liver	White Carneau Pigeon	14 years	Male	Cutaneous Leiomyosarcomas	Cervical tissue	Desmin (Positive) α‐SMA (Positive) Vimentin (Positive) S100 (Negative) Pancytokeratin (Negative) von Willebrand (Negative)	(Fabian et al. 2021)
Kidney, brain, liver	African Goose	10 months	Male	Leiomyosarcoma		α‐SMA (Positive) Vimentin (Positive)	(Silva et al. 2021)
Liver	Broiler Chicken	57 days	Female	Myxoid Leiomyosarcoma	Gizzard	Desmin (Positive) α‐SMA (Positive) Actin (Positive)	(Sato et al. 2002)
Liver, kidney	Sarus Crane	12 years	Female	Myxoid Leiomyosarcoma	Subcutaneous tissue of the Tarsometatarsus	Muscle Actin (positive) Vimentin (Positive) Smooth Muscle Myosin (Positive)	(Frazier et al. 1993)
Liver, spleen, kidneys, heart, lungs	Larry Breed Hen	6 years	Female	Leiomyosarcoma	Leg	Desmin (Positive) α‐SMA (Positive) Vimentin (Positive) Myogenin (Negative)	(Kheirandish et al. 2016)
Liver	Rose‐ringed parakeet	3 years	Female	Leiomyosarcoma	Leg	α‐SMA (Positive) Myogenin (Positive) Desmin (Positive)	(Azizi et al. 2026)

Abbreviation: NS, not specified.

## 4 Conclusion

This report presents what we believe to be the first documented case of LMS in a budgerigar, with metastases observed in the bones and kidneys. It emphasizes the importance of early diagnosis, which may help reduce the risk of metastasis and enable timely treatment to decrease mortality rates. However, it is crucial to note that these conclusions are based on a single case and cannot be generalized. The limited treatment options available for avian species and the specific nature of this case constrain definitive management recommendations. Therefore, further research involving larger groups of budgerigars is needed to enhance our understanding of the epidemiology, risk factors, and clinical features of LMS in these birds.

## Author Contributions

**Hemad Shafiei**: data curation, funding acquisition, investigation, methodology, visualization, writing – original draft, supervision, project administration, validation, and visualization. **Reza Kheirandish**: pathology finding preparation, conceptualization, funding acquisition, investigation, methodology, project administration. **Shahriar Dabiri**: pathology finding preparation, conceptualization, funding acquisition, investigation, methodology, project administration. **Hamzeh Soltani**: writing – original draft, conceptualization, data curation and formal analysis. **Ali Keshavarzrad**: writing – original draft, conceptualization, data curation and formal analysis. All the authors approved the final version of this manuscript.

## Funding

The authors have nothing to report.

## Ethics Statement

The authors confirm that the ethical policies of the journal, as noted on the journal's author guidelines page, have been adhered to and the appropriate ethical review committee approval has been received. The trial convention was affirmed by the animal welfare committee (which covered IACUC approval) of the Faculty of Veterinary Medicine, Shahid Bahonar University of Kerman, Kerman, Iran.

## Conflicts of Interest

The authors declare no conflicts of interest.

## Data Availability

The data used to support the findings of this study are available from the corresponding author upon request.
